# Coin Aspiration Presenting as Chronic Cough and Tracheoesophageal Fistula

**DOI:** 10.7759/cureus.50283

**Published:** 2023-12-10

**Authors:** Harsh Kothari, Aravinth Prasanth Jawahar, Aditya Badheka, Madhuradhar Chegondi

**Affiliations:** 1 Pediatric Critical Care Medicine, Dayton Children's Hospital, Dayton, USA; 2 Pediatric Critical Care Medicine, Stead Family Children's Hospital, University of Iowa Hospitals and Clinics, Iowa City, USA

**Keywords:** esophageal foreign body, rigid bronchoscopy, chronic cough, tracheo-esophageal fistula, tracheobronchial foreign body aspiration

## Abstract

Chronic cough can be a diagnostic challenge in the pediatric population. Foreign body aspiration without typical signs and symptoms can often be overlooked as a cause of chronic cough in children. Coin aspirations in the trachea typically have a sagittal orientation on an anteroposterior (AP) chest radiograph. We report a rare case of a previously healthy five-year-old girl presenting with a chronic cough for five months caused by a coin with a coronal orientation on an AP chest radiograph. The coin, initially presumed to be lodged in the esophagus, was actually lodged in the cervical trachea, leading to the development of a tracheoesophageal fistula (TEF). Her AP chest radiograph showed a coronal, circular radio-opaque shadow and the lateral view a tangential radio-opaque shadow, prompting an initial evaluation by esophagogastroduodenoscopy, which was normal. She then underwent rigid bronchoscopy, revealing a coin lodged in the trachea along with a TEF. Surgical removal was achieved through an external approach with a vertical tracheotomy and insertion of a tracheostomy tube. Five days later, a repeat rigid bronchoscopy showed a well-healed TEF, and she was successfully decannulated. She was ultimately discharged home on room air and oral feeds. TEF as a complication of a foreign body lodged in the trachea or esophagus is rare but life-threatening. Foreign body aspiration should always be considered in the differential diagnosis when evaluating younger children with chronic cough.

## Introduction

Chronic cough in children is defined as a daily cough of at least four weeks' duration [1]. There are several etiologies for chronic cough in children, which can pose a diagnostic challenge. One of the most important causes of chronic cough is foreign body aspiration (FBA). The prevalence of nonfatal FBA cases is about 20.4 per 100,000 children under the age of 14, and that of fatal FBA cases is 0.43 per 100,000 children under the age of five [2]. In the absence of typical signs and symptoms, FBA as a cause for chronic cough can easily be overlooked. A retained foreign body in the respiratory tract can lead to serious complications such as bronchiectasis, emphysema, and pneumonia [3]. Coin aspirations in the trachea have a sagittal orientation and appear tangential on frontal radiographs and en face on lateral radiographs [4]. We report an unusual case of a five-year-old girl with tracheal coin aspiration, but with a coronal orientation on an anteroposterior (AP) chest radiograph, complicated by the development of a tracheoesophageal fistula (TEF). She presented with chronic intermittent cough for five months, and on an AP chest radiograph, there was evidence of a coronal, circular radiopaque shadow. This necessitated emergent evaluation by esophagogastroduodenoscopy (EGD), which was normal, as the coin was initially thought to be in the esophagus. An airway evaluation by rigid bronchoscopy showed a coin lodged in the trachea along with a TEF. The coin was then surgically removed through an external approach with a vertical tracheotomy and insertion of a tracheostomy tube. The consent to publish this case report was obtained from the patient’s parents.

## Case presentation

A five-year-old girl was brought to the emergency department (ED) by her parents with complaints of a dry cough for five months that had acutely worsened over the last 24 hours, with no immediate precipitating events. The parents reported that the cough was worse upon lying supine and in the morning and was not associated with wheezing, shortness of breath, or hemoptysis; they denied any concerns for FBA. Her past medical history is significant for dental caries, for which she had undergone multiple dental procedures. She was born full-term and has no neurological, developmental, or feeding abnormalities and no known allergies. She was evaluated twice by her primary care physician and once in an urgent care and was prescribed loratadine, with no improvement in symptoms. No laboratory or radiographic investigations were recommended. On the day of presentation, she had acutely worsened with increased work of breathing while at school, necessitating a transfer and evaluation in the ED. On arrival, she was noted to be alert, active, and in no respiratory distress, hemodynamically stable with oxygen saturations >92% on ambient air. The remainder of her physical examination was benign.

**Figure 1:**
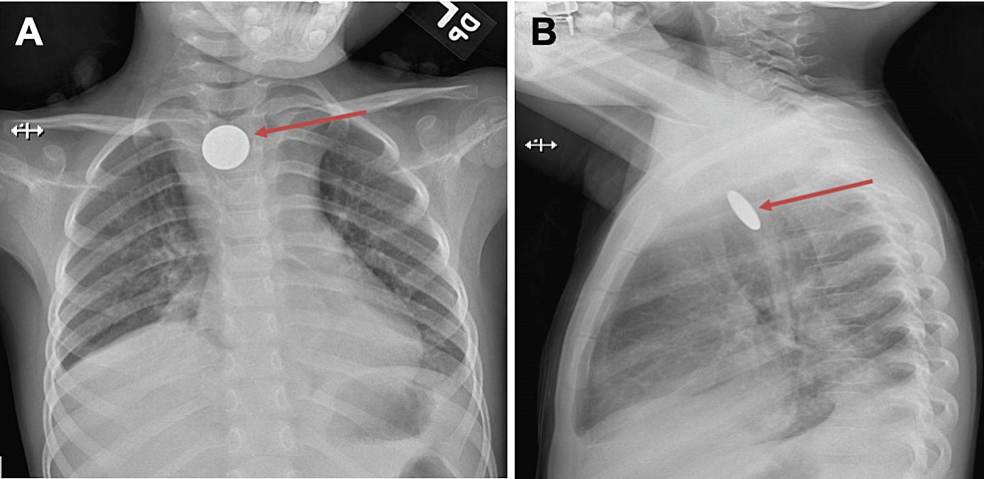
Chest X-ray anteroposterior (A) and lateral view (B) showing a radio-opaque shadow (red arrow) in the upper central thoracic region oriented in a coronal and tangential position, respectively.

**Figure 2:**
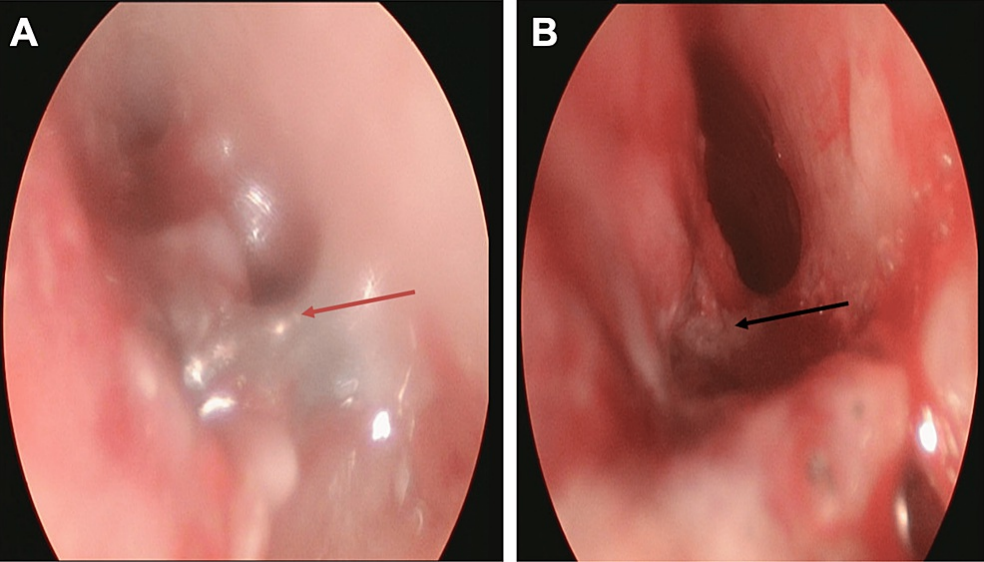
Rigid bronchoscopy of the trachea (A) showing a coin (red arrow) lodged in the cervical trachea in a coronal position with surrounding copious purulent secretions and blood and a view of the trachea (B) after removal of the foreign body, revealing a tracheoesophageal fistula and granulation tissue (black arrow) along the posterior wall of the trachea.

**Figure 3:**
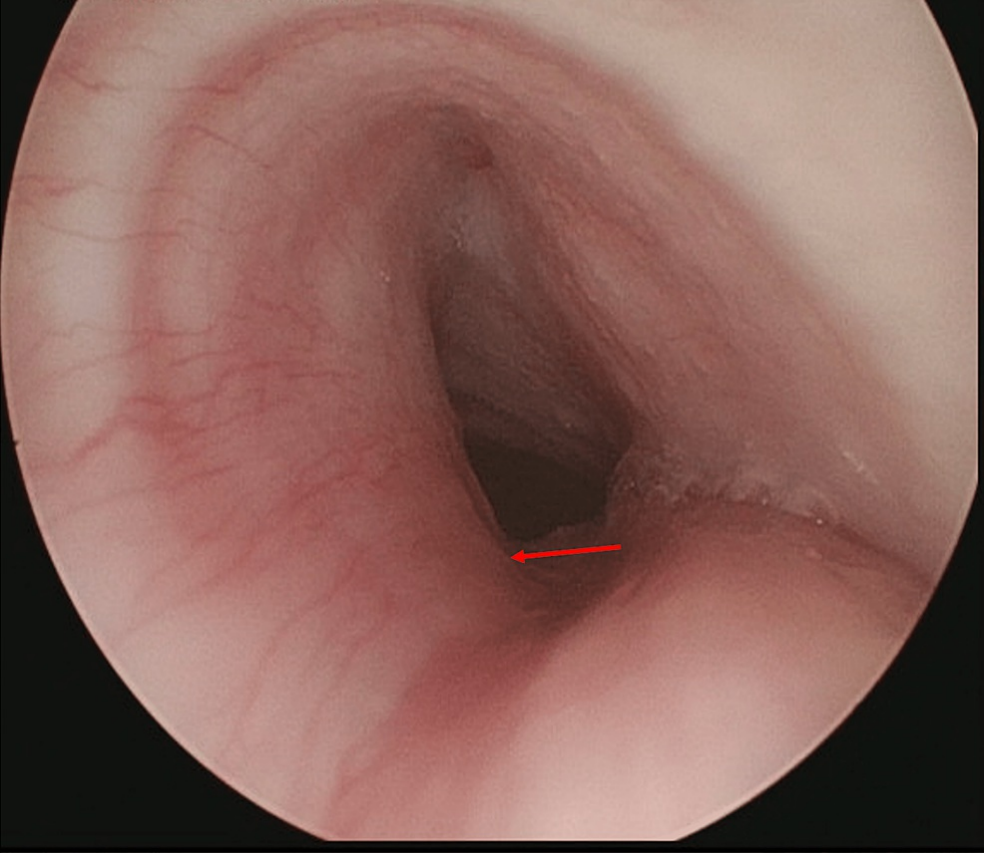
Rigid bronchoscopy of the trachea on postoperative day 21 showing the healed tracheoesophageal fistula site (red arrow) and resolution of granulation tissue.

An AP chest radiograph (Figure 1A) showed a coronal, circular radio-opaque shadow, appearing as a tangential shadow on a lateral view (Figure 1B). This circular radio-opaque shadow, likely a coin, was thought to be in the proximal esophagus. The child underwent an emergent EGD under general anesthesia with no evidence of a foreign body and no other abnormal findings. A repeat C-arm chest radiograph in the operating room showed persistence of the radio-opaque shadow similar to the initial radiograph, raising concern for a foreign body in the airway. The pediatric otolaryngology team evaluated the airway with a rigid bronchoscope and found a coin lodged in the cervical trachea in a coronal position with copious purulent secretions (Figure 2A). A three-mm TEF with granulation tissue was also noted at the site of foreign body lodgment (Figure 2B). Unable to remove the coin endoscopically through micro forceps due to its size and coronal orientation, an external approach through vertical tracheotomy was required to remove the foreign body successfully. Subsequently, a tracheostomy tube and nasogastric feeding tube were placed. She was also prescribed a seven-day course of intravenous antibiotics. On postoperative day (POD) 5, direct laryngoscopy and bronchoscopy (DLB) showed a healed fistula, and she was decannulated successfully. The repeat DLB on POD 21 showed 40% luminal obstruction due to anterior tracheal wall collapse from the prior tracheostomy site but no evidence of recurrence of the TEF (Figure 3), later confirmed on a barium swallow study. The child was discharged home on room air and on oral feeds. At a scheduled six-week follow-up visit, a rigid bronchoscopy evaluation of the trachea showed no evidence of recurrence of the TEF or subsequent tracheal stenosis as a complication.

## Discussion

Chronic cough in the pediatric population is defined as a daily cough for four or more weeks [1]. It has an estimated prevalence of about 5 to 10% with a significant disease burden to patients and their parents [5,6]. Identifying the correct diagnosis for chronic cough can be an arduous task for the physician, as the differential diagnosis is broad. Current guidelines emphasize the importance of a detailed medical history and physical examination in making the diagnosis of FBA [1,7]. Multiple factors can contribute to a delayed or missed diagnosis of FBA. Mu et al., in a retrospective review of 210 cases of FBA, identified parental negligence as the most common cause, followed by misdiagnosis by medical professionals. It is recommended to obtain a chest radiograph and spirometry (if age-appropriate) in cases of nonspecific chronic cough [3]. FBA should always be considered, especially in a younger child presenting with nonspecific chronic cough. Pediatric FBA is a significant healthcare concern with an estimate of more than 1900 annual hospital admissions [8]. Usually, the presentation of FBA is acute, with a median time after an aspiration event reported as one day (interquartile range of 0-4 days), with foreign bodies located in the main bronchus (78%) or primary/secondary bronchi (20%) [9]. Food products account for 67% of foreign bodies [9]. Reports on long-standing FBA (LSFBA) show the median time to presentation is four weeks (mean 8.8 weeks), with non-productive cough (86%) and dyspnea (51%) as the most common presenting symptoms [10]. About 83% of children with LSFBA had a foreign body dislodged in either of the main bronchi [10]. Nuts or other organic material was found in 63% of children with LSFBA [10]. LSFBA with a coin is extremely rare, and there are very few case reports available [11]. Su et al. reported LSFBA with a retained coin for over 30 years [12]. The chest radiograph is usually diagnostic of a coin aspiration and indicates the location of the coin in the trachea or esophagus. Coins in the trachea are typically oriented sagittally and appear tangential on frontal radiographs and en face on lateral radiographs [4]. The presence of a coronal circular radio-opaque shadow on an AP chest radiograph, appearing as a tangential radio-opaque shadow on a lateral view, prompted an initial evaluation with an EGD in our patient, as the coin was presumed to be in the esophagus. Coins in the trachea, oriented in a coronal position in an AP chest radiography, are very uncommon. We hypothesize that the foreign body in the trachea could have changed orientation due to chronicity and the subsequent fistulous connection, along with granulation tissue and copious secretions.

Our patient’s clinical course with the development of an acquired TEF demonstrates a rare but life-threatening complication of LSFBA [13]. Most reported cases describe a foreign body in the esophagus as the likely origin of the foreign body-related TEF [13]. A foreign body in the trachea leading to a TEF, as in our index patient, is extremely rare, and our literature search yielded only one other case report describing the development of a TEF secondary to a tracheal foreign body, which required surgical repair [14].

## Conclusions

Chronic cough in the pediatric population can be a diagnostic challenge, given the broad list of differential diagnoses that can be considered. Evaluating providers should have a high index of suspicion for FBA in young children even if the history and physical examination are inconclusive and investigate further with a chest radiograph with AP and lateral views in patients presenting with non-specific chronic cough. However, the orientation of the radio-opaque shadow on chest radiograph cannot be entirely relied upon to pinpoint the location of the foreign body as in our index patient with a tracheal coin that was initially thought to be in the esophagus. Acquired TEF is an uncommon but a life-threatening complication of LSFBA.
